# Glycaemic Control by Sociodemographic Factors in Adults With Type 1 Diabetes in England: Trends From 2007–2008 to 2023–2024

**DOI:** 10.1111/dom.70929

**Published:** 2026-06-01

**Authors:** Muhammad Saleem Khan, Jonathan Valabhji, Naveed Sattar, Helen Murphy, Fulya Mehta, Partha Kar, Clare Hambling, Kamlesh Khunti, Garry D. Tan, Naomi Holman

**Affiliations:** ^1^ School of Public Health Imperial College London London UK; ^2^ Department of Metabolism, Digestion and Reproduction, Faculty of Medicine, Chelsea and Westminster Hospital Campus Imperial College London London UK; ^3^ School of Cardiovascular and Metabolic Health University of Glasgow Glasgow UK; ^4^ Norwich Medical School University of East Anglia Norwich UK; ^5^ Alder Hey Children's NHS Foundation Trust Liverpool UK; ^6^ Portsmouth Hospitals University NHS Trust Portsmouth UK; ^7^ Litcham Health Centre Norfolk UK; ^8^ Diabetes Research Centre, College of Life Sciences Leicester General Hospital, University of Leicester Leicester UK; ^9^ Oxford Centre for Diabetes, Endocrinology and Metabolism Oxford University Hospitals Foundation Trust Oxford UK; ^10^ NIHR Oxford Biomedical Research Centre Oxford UK; ^11^ School of Population Health Royal College of Surgeons in Ireland Dublin Ireland

**Keywords:** glycaemic control, observational study, real‐world evidence, Type 1 diabetes

## Abstract

**Aims:**

This study evaluated trends in glycaemic control in adults with Type 1 diabetes in England from 2007–2008 to 2023–2024, stratified by age, sex, ethnicity and socioeconomic deprivation.

**Materials and Methods:**

Adults aged ≥ 20 years from the National Diabetes Audit were analysed. Trends in mean HbA1c were assessed using linear mixed‐effects models. Logistic regression examined associations between sociodemographic factors and having an HbA1c ≤ 58 mmol/mol (≤ 7.5%); odds ratios were converted to relative risks.

**Results:**

In 2007–2008, 32.6% of 133 035 adults had an HbA1c ≤ 58 mmol/mol, increasing to 39.1% of 226 005 adults in 2023–2024. Mean HbA1c declined from 68.8 (95% CI: 68.6–69.0) to 66.2 mmol/mol (95% CI: 66.1–66.3). By age, the largest improvement occurred in those aged 20–24 years, from 76.7 to 70.1 mmol/mol (*p* < 0.001), while adults > 64 years saw a non‐significant increase from 63.7 to 63.8 mmol/mol (*p* = *0.203*). Women consistently had higher mean HbA1c than men (2023–2024: 66.1 vs. 65.8 mmol/mol), declining from 69.4 to 66.1 mmol/mol in women and from 68.4 to 65.8 mmol/mol in men (both *p* < 0.001) and a lower adjusted probability of threshold attainment (38.4% [95% CI: 38.0–39.4] vs. 38.7% [95% CI: 38.2–39.3]). Marked ethnic inequalities persisted in 2023–2024: mean HbA1c was highest in Black (72.1 mmol/mol, 95% CI: 71.5–72.7) and Asian adults (69.8 mmol/mol, 95% CI: 69.4–70.2) compared with White adults (65.3 mmol/mol, 95% CI: 65.1–65.5). Socioeconomic gradients remained substantial, with threshold attainment at 46.4% (95% CI: 45.6–47.2) in the least deprived versus 32.3% (95% CI: 31.4–33.9) in the most deprived group.

**Conclusions:**

Although HbA1c improved overall, inequalities by age and deprivation widened, highlighting the need for equitable targeted care.

## 1 Introduction

Type 1 diabetes is a lifelong autoimmune condition requiring exogenous insulin due to pancreatic beta‐cell destruction [1], affecting over 271 000 adults in England and Wales [2, 3]. It poses significant clinical challenges because of complex management needs, risk of acute and chronic complications and substantial healthcare costs [4, 5].

Glycaemic control, as measured by glycated haemoglobin (HbA1c), remains a cornerstone of Type 1 diabetes management.

Studies have shown that younger age, female sex, non‐White ethnicity and lower socioeconomic status are significantly associated with poorer glycaemic control in adults with Type 1 diabetes [6, 7, 8]. Studies consistently report higher HbA1c among females than males, potentially mediated by hormonal fluctuations, differential healthcare engagement and psychosocial stressors [8, 9]. Ethnic inequalities in diabetes care, particularly among South Asian and Black populations, are a significant concern due to cultural beliefs, socioeconomic disadvantage, health literacy gaps and healthcare access barriers [10]. Previous studies have shown that socioeconomic deprivation correlates with poorer Type 1 diabetes outcomes, such as higher hospital admission rates [11], suboptimal glycaemic control [11] and increased cardiometabolic risk factors [12]. However, longitudinal evidence examining how these inequalities have evolved over recent years in England remains limited.

The National Diabetes Audit (NDA) offers a comprehensive dataset for evaluating diabetes care in England [13]. This study aimed to evaluate trends in glycaemic control in adults with Type 1 diabetes in England and how they vary by age, sex, ethnicity and social deprivation.

## 2 Materials and Methods

This study employed a retrospective sequential cohort design using data from the NDA, which has collected data on individuals with diabetes registered with primary or specialist healthcare providers in England since 2003. Each audit period in the NDA spans 15 months, from 1 January of the first year to 31 March of the subsequent year. Detailed descriptions of the dataset have been reported previously [13]. Data for this analysis included people registered with healthcare providers in England between 2007–2008 and 2023–2024. The study population included all adults aged 20 years and older with a recorded diagnosis of Type 1 diabetes. Type 1 diabetes was identified based on the latest reported coded type of diabetes from specialist care in the NDA. If no data have been received from specialist care, then the latest diagnostic code recorded in primary care was used to identify the type of diabetes.

The primary outcome was glycaemic attainment, defined as the proportion of adults with Type 1 diabetes with an HbA1c ≤ 58 mmol/mol (< 7.5%) and as the mean HbA1c (mmol/mol). The HbA1c threshold of ≤ 58 mmol/mol (7.5%) was chosen because it is the established national audit metric used by the Quality and Outcomes Framework (QOF) and reported by the NDA [14]. This threshold provides a practical benchmark for balancing long‐term risks of microvascular complications with the acute risk of severe hypoglycaemia in a large, heterogeneous adult population with Type 1 diabetes. Using this standardised threshold allows comparability across audit years and supports evaluation of trends in glycaemic control at the population level.

The explanatory socio‐demographic variables included age (completed years), sex, ethnicity and social deprivation. Age was categorised into four groups (20–24, 25–39, 40–64 and > 64 years) to explore variations in glycaemic control across the adult lifespan. Sex (male/female) was included to evaluate sex‐specific trends. Ethnicity was classified according to standard National Health Service (NHS) categories and grouped into White, Mixed, Asian, Black, other and not stated. Social deprivation was assessed using the Index of Multiple Deprivation (IMD) at the Lower Layer Super Output Area (LSOA) level and categorised into quintiles (1 = most deprived, 5 = least deprived) [15].

### 2.1 Statistical Analysis

All statistical analyses were performed using *R* 4.4.1 [16]. Glycaemic control outcomes were assessed from 2007–2008 to 2023–2024, measuring the proportion of adults with Type 1 diabetes meeting the HbA1c threshold (≤ 58 mmol/mol) and mean HbA1c (mmol/mol) for the entire cohort for whom HbA1c was available. Outcomes were stratified by age group, sex, ethnicity and IMD quintile. For each audit period, we calculated the proportion of individuals meeting the HbA1c threshold (≤ 58 mmol/mol [< 7.5%]) and the mean HbA1c (mmol/mol) in each audit period, providing a trend of glycaemic control.

Multivariable logistic regression was used to assess the temporal trend in the associations between sociodemographic factors and the likelihood of meeting the HbA1c threshold across all audit years (2007–2008 to 2023–2024).

Stratified logistic regression models were used to estimate changes in the likelihood of meeting the HbA1c threshold between 2007–2008 (reference) and 2023–2024 within each sociodemographic subgroup. Audit year was included as a covariate in each model, with 2007–2008 as the reference period, providing an estimate of change in the likelihood of meeting the HbA1c threshold between 2007–2008 and 2023–2024. Each model was adjusted for the remaining sociodemographic variables (i.e., age, sex, ethnicity and IMD quintile). Adjusted odds ratios (ORs) were converted to relative risks (RRs) with 95% confidence intervals (CIs) using the baseline probabilities of HbA1c control in the 2007–2008 audit period for each corresponding category, which were then converted into a percentage change to enhance clinical interpretability.

In a sensitivity analysis, we changed the denominator from adults with a valid HbA1c measurement to all adults with Type 1 diabetes to explore the potential impact of excluding those with missing HbA1c data.

To evaluate changes in mean HbA1c over time, we employed linear mixed‐effects models. Linear mixed‐effects models were fitted with random intercepts for individuals, adjusting for age, sex, ethnicity, IMD quintile and audit year. Each model was adjusted for the remaining sociodemographic variables. These models accounted for individual‐level variability and repeated measures by incorporating random intercepts for each person. The fixed effects included audit period, age, sex, IMD quintile and ethnicity, with the audit period 2007–2008 serving as the baseline for comparison. We report absolute adjusted mean HbA1c in subsequent audit periods relative to 2007–2008, along with 95% CIs, which are represented as dispersion bars in all figures.

Statistical significance was defined as *p* < 0.05 for all analyses. All *p* values refer to comparisons with the 2007–2008 reference period, conducted separately within each stratum of a variable. Analyses were performed using complete case analysis, including only records with non‐missing values for all variables of interest. This approach was deemed appropriate as the proportion of missing data was low, minimising potential bias.

### 2.2 Information Governance

NDA data is collected and used in line with NHS England's purposes as required under the statutory duties outlined in the NHS Act 2006 and the Health and Social Care Act 2012. There is controlled data access by appropriately approved individuals on secure data environments entirely within the NHS England infrastructure. Data is processed for specific purposes only, including operational functions, service evaluations and service improvement. The data used to produce this analysis has been disseminated within NHS England under Directions issued under Section 254 of the Health and Social Care Act 2012. Ethics committee approval is not required for these specific purposes. All numbers taken from the NDA are rounded to the nearest 5 to protect individuals' confidentiality.

## 3 Results

In 2007–2008, there were 155 730 adults aged ≥ 20 years with Type 1 diabetes in England, of whom 133 035 (85.4%) had a recorded HbA1c value; in 2023–2024, the corresponding figures were 259 660 and 226 005 (87.0%), respectively. The percentage of General Practitioner (GP) practices participating in the audit had significantly increased over time (Figure S1). The participation rate was relatively low in the early years, with a notable dip to 54.9% in 2013–2014 and 2014–2015. However, it has since seen a rise, maintaining a very high level of over 97% since 2018–2019. Table 1 compares the mean HbA1c by demographic characteristics in adults with Type 1 diabetes in England between 2007–2008 and 2023–2024.

**TABLE 1:** Comparison of mean HbA1c by demographic characteristics in adults with Type 1 diabetes in England between 2007–2008 and 2023–2024.

Characteristic	Audit period 2007–2008 (*n* = 133 035)		Audit period 2023–2024 (*n* = 226 005)		*p*
	*n*	Mean (SD)	*n*	Mean (SD)	
Age					
20–24 years	7295	76.7 (23.2)	14 545	70.1 (21.9)	< 0.001
25–39 years	33 410	70.7 (20.1)	59 590	66.4 (20.0)	< 0.001
40–64 years	64 705	69.1 (18.0)	107 150	66.0 (17.6)	< 0.001
> 64 years	27 630	63.7 (16.0)	44 730	63.8 (15.3)	0.203
Sex					
Male	74 450	68.4 (18.5)	127 060	65.8 (18.3)	< 0.001
Female	58 585	69.4 (19.0)	98 945	66.1 (18.1)	< 0.001
Ethnicity					
White	117 700	68.7 (18.4)	196 375	65.6 (17.8)	< 0.001
Mixed	1070	70.3 (21.5)	2760	68.8 (21.5)	0.050
Asian	6480	70.3 (20.7)	11 045	67.7 (19.6)	< 0.001
Black	4260	69.9 (23.2)	8280	70.0 (22.9)	0.812
Other	2815	68.1 (19.8)	6130	65.6 (19.0)	< 0.001
Not stated	710	65.8 (17.8)	775	63.1 (17.4)	0.003
IMD quintile					
Q1 most deprived	27 395	71.3 (21.0)	44 265	70.4 (20.6)	< 0.001
Q2	26 085	69.9 (19.6)	44 485	67.4 (18.9)	< 0.001
Q3	26 580	68.6 (18.1)	45 775	65.6 (17.7)	< 0.001
Q4	26 225	67.6 (17.5)	45 350	64.0 (16.7)	< 0.001
Q5 least deprived	25 850	66.6 (16.7)	42 770	62.3 (15.7)	< 0.001

Abbreviations: IMD = index of multiple deprivation, SD = standard deviation.

Mean HbA1c declined in most age groups between 2007–2008 and 2023–2024, with the most substantial reduction observed in those aged 20–24 years (76.7 to 70.1 mmol/mol; *p* < 0.001). In contrast, mean HbA1c levels for adults aged 65 years and older remained stable over the study period (63.7 mmol/mol in 2007–2008 vs. 63.8 mmol/mol in 2023–2024; *p* = 0.203). Males showed a decline from 68.4 to 65.8 mmol/mol, *p* < 0.001; females from 69.4 to 66.1 mmol/mol, *p* < 0.001. By ethnicity, HbA1c declined in both the White population (68.7 to 65.6, *p* < 0.001) and the Asian population (70.3 to 67.7 mmol/mol, *p* < 0.001). People in the most deprived quintile had consistently higher mean HbA1c (71.3 to 70.4 mmol/mol) than those in the least deprived quintile (66.6 to 62.3 mmol/mol, *p* < 0.001 *for change over time*).

**FIGURE 1:**
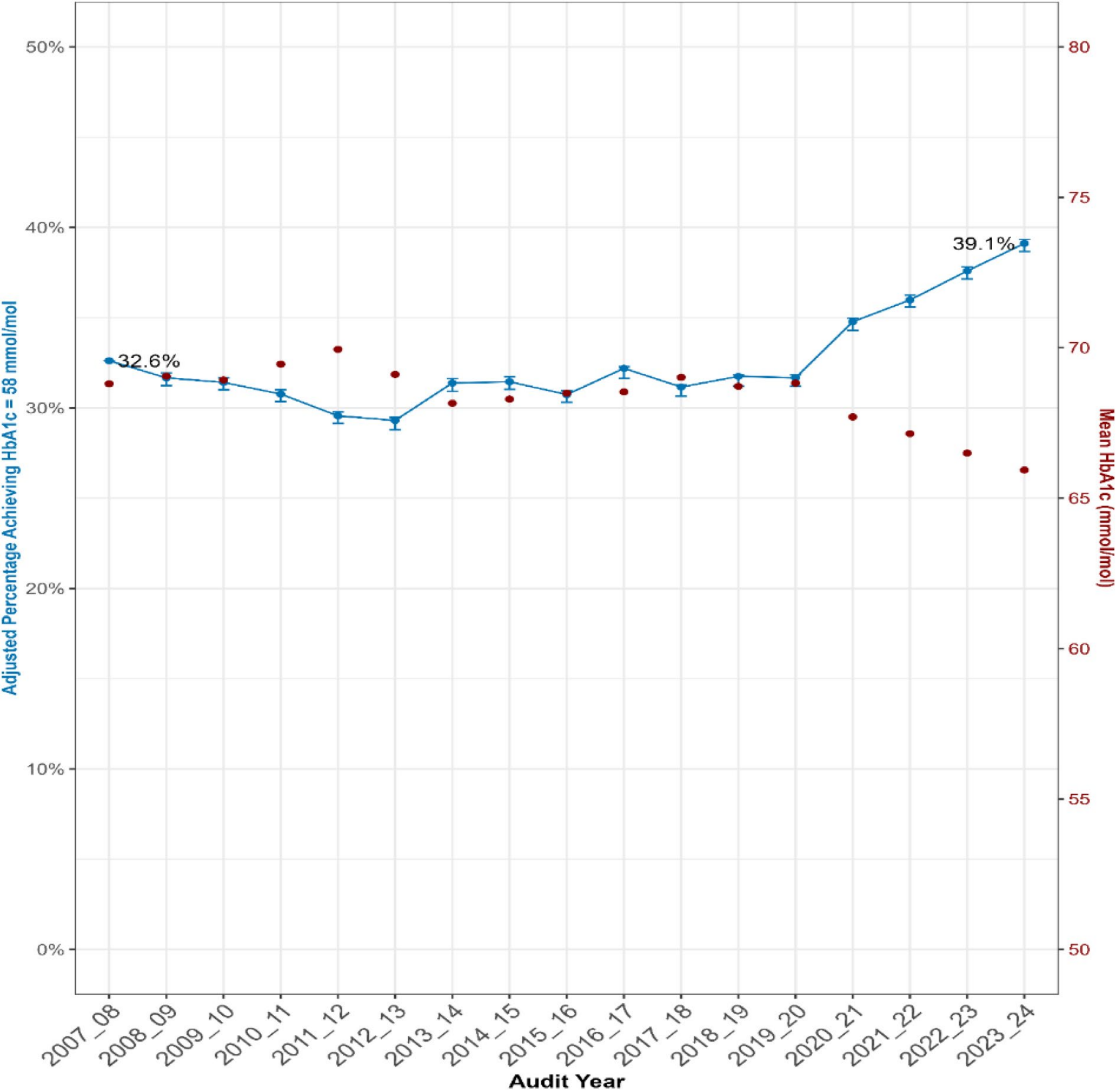
Trends in the adjusted percentage of adults with Type 1 diabetes in England with HbA1c ≤ 58 mmol/mol and in mean HbA1c, from 2007–2008 to 2023–2024. Adjusted for age, sex, ethnicity and deprivation. Audit period 2007–2008 is the baseline reference; all estimates compare subsequent periods to baseline.

Figure 1 illustrates trends in the adjusted percentage of adults with Type 1 diabetes in England with an HbA1c ≤ 58 mmol/mol, alongside mean HbA1c, from 2007–2008 to 2023–2024. Mean HbA1c remained stable during the early years of the study period, moving from 68.8 mmol/mol in the 2007–2008 reference year to 69.8 mmol/mol (95% CI: 68.7–69.9) in 2012–2013. Following this initial period of stability, a gradual decline was observed, which transitioned into a subsequent plateau. A more pronounced reduction emerged after 2020–2021, with mean HbA1c falling to 66.2 mmol/mol (95% CI: 66.1–66.3) by 2023–2024. The adjusted percentage meeting the HbA1c threshold initially declined from 32.6% (95% CI: 31.9%–33.3%) in 2007–2008 to a low of approximately 29.0% (95% CI: 28.3%–29.7%) in 2012–2013. Subsequently, a steady increase in the percentage of people with an HbA1c ≤ 58 mmol/mol was observed, reaching 39.1% (95% CI: 38.3%–39.9%) by 2023–2024.

**FIGURE 2:**
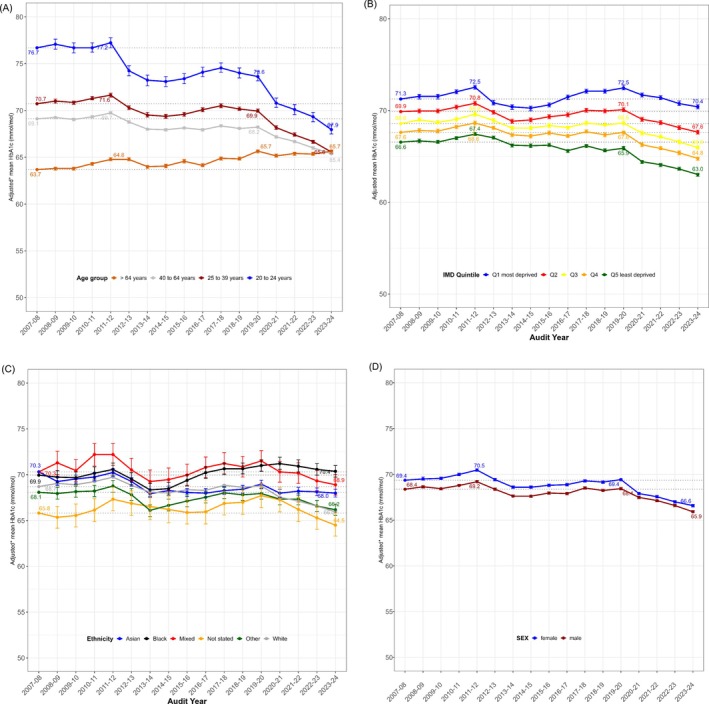
(A) Adjusted mean HbA1c trends by age group in English Adults with Type 1 diabetes, 2007–2008 to 2023–2024. Adjusted for sex, ethnicity and deprivation. Audit period 2007–2008 is the baseline reference; all estimates compare subsequent periods to baseline. (B) Adjusted mean HbA1c trends by IMD in English Adults with Type 1 diabetes, 2007–2008 to 2023–2024. Adjusted for age, sex and ethnicity. (C) Adjusted mean HbA1c trends by ethnicity in English Adults with Type 1 diabetes, 2007–2008 to 2023–2024. Adjusted for age, sex and IMD. (D) Adjusted mean HbA1c trends by sex in English Adults with Type 1 diabetes, 2007–2008 to 2023–2024. Adjusted for age, ethnicity and IMD.

**FIGURE 3:**
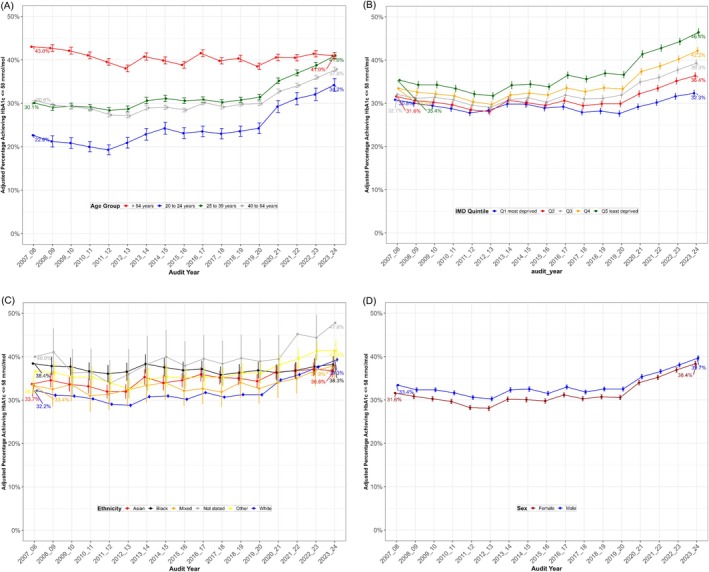
(A) Trends in the adjusted percentage of adults with Type 1 diabetes in England with HbA1c ≤ 58 mmol/mol, stratified by age group from 2007–2008 to 2023–2024. Adjusted for sex, ethnicity and deprivation. (B) Trends in the adjusted percentage of adults with Type 1 diabetes in England with HbA1c ≤ 58 mmol/mol, stratified by IMD quintile from 2007–2008 to 2023–2024. Adjusted for age, sex and ethnicity. (C) Trends in the adjusted percentage of adults with Type 1 diabetes in England with HbA1c ≤ 58 mmol/mol, stratified by ethnicity from 2007–2008 to 2023–2024. Adjusted for age, sex and IMD. (D) Trends in the adjusted percentage of adults with Type 1 diabetes in England with HbA1c ≤ 58 mmol/mol, stratified by sex from 2007–2008 to 2023–2024. Adjusted for age, ethnicity and IMD.

Figure 2 shows trends in adjusted mean HbA1c among adults with Type 1 diabetes in England from 2007–2008 to 2023–2024, stratified by age group (Figure 2A), IMD quintile (Figure 2B), ethnicity (Figure 2C) and sex (Figure 2D). In the 20–24‐year age group, adjusted mean HbA1c was 76.7 mmol/mol in 2007–2008 (reference year), decreasing to 67.9 mmol/mol in 2023–2024 (95% CI: 67.41–68.41, *p* < 0.001). Among adults aged 65 years and older, adjusted mean HbA1c increased slightly from 63.7 mmol/mol in 2007–2008 to 65.6 mmol/mol in 2023–2024 (95% CI: 65.47–65.78, *p* < 0.001) (Figure 2A). People living in the most deprived IMD quintile had an adjusted mean HbA1c of 71.3 mmol/mol in 2007–2008 and remained high at 70.4 mmol/mol in 2023–2024 (95% CI: 70.18–70.61, *p* < 0.001). In comparison, those living in the least deprived quintile of neighbourhoods showed a decline in adjusted mean HbA1c from 66.6 mmol/mol in 2007–2008 to 63.0 mmol/mol in 2023–2024 (95% CI: 62.82–63.23, *p* < 0.001) (Figure 2B). Following the COVID‐19 period, improvements in adjusted mean HbA1c were less pronounced among Asian and Black adults compared to other ethnic groups (Figure 2C). In 2023–2024, the adjusted mean HbA1c remained elevated at 70.4 mmol/mol in Black adults, representing no significant change from the 2007–2008 baseline (*p* = 0.163 for change over time). In contrast, while the mean HbA1c for Asian adults fell to 68.0 mmol/mol, this represented a statistically significant, although modest, longitudinal reduction (*p* = 0.001 for change over time). These findings highlight the persistence of ethnic inequalities in glycaemic control. A consistent sex‐based difference in adjusted mean HbA1c was maintained throughout the study period, with females exhibiting slightly higher levels than males (Figure 2D). Compared to the 2007–2008 baseline, both groups showed significant longitudinal declines by 2023–2024; mean HbA1c fell to 66.6 mmol/mol in females (*p* < 0.001 for change over time) and to 65.9 mmol/mol in males (*p* < 0.001 for change over time). Figure 3 shows trends in adjusted percentage of adults with Type 1 diabetes with a HbA1c ≤ 58 mmol/mol in England from 2007–2008 to 2023–2024, stratified by age group (Figure 3A), IMD quintile (Figure 3B), ethnicity (Figure 3C) and sex (Figure 3D). The > 64 years age group consistently maintained the highest adjusted percentage, starting at 43.0% in 2007–2008, before ending at 40.9% (95% CI: 40.2%–41.7%, *p* < 0.001) in 2023–2024. Although the proportion meeting this threshold declined over time, older adults remained more likely than all younger age groups to reach HbA1c ≤ 58 mmol/mol (≤ 7.5%) throughout the study period. The 20–24 years group, despite starting lowest (22.6%), showed the greatest improvement, rising to 34.2% (95% CI: 32.7%–35.7%, *p* < 0.001) by 2023–2024 (Figure 3A). People living in the least deprived quintile consistently showed the highest percentage with an HbA1c ≤ 58 mmol/mol, rising from 35.4% in 2007–2008 to 46.4% (95% CI: 45.6%–47.2%, *p* < 0.001) in 2023–2024; whereas the most deprived group consistently had the lowest percentage meeting this threshold with no change between 2007–2008 (32.1%, 95% CI: 31.6%–33.1%) and 2023–2024 (32.3%, 95% CI: 31.4%–33.9%) (Figure 3B). The White ethnic group's adjusted percentage meeting the threshold rose from 32.2% in 2007–2008 to 39.3% (95% CI: 38.9%–39.7%) by 2023–2024. The Asian group began similarly at around 33.7% in 2007–2008 but ended slightly lower at 36.8% (95% CI: 35.31%–38.42%, *p* < 0.001) by 2023–2024, showing a more modest increase compared to the White ethnic group over the period. Other ethnic groups exhibited varied proportions achieving HbA1c ≤ 58 mmol/mol (Figure 3C). The *p* values reflect comparisons within each sex separately, using 2007–2008 as the reference period. Therefore, for men, each subsequent audit year is compared with men in 2007–2008, and similarly for women, each subsequent audit year is compared with women in 2007–2008. There was a consistent difference by sex in the adjusted percentage of participants meeting the treatment threshold of HbA1c during the study period, with females showing a slightly lower percentage than males; in 2023–2024, the percentage was 38.4 (*p* < 0.001 for change over time) in females compared to 38.7 (*p* < 0.001 for change over time) in males (Figure 3D).

Results from the sensitivity analysis, which included all adults regardless of HbA1c data completeness, were consistent with the main findings (Figure S4). The adjusted percentage of adults with Type 1 diabetes meeting the HbA1c threshold was 27.9% in 2007–2008, increasing to 34.8% (95% CI: 34.5%–35.1%) in 2023–2024 (Figure S4A). When stratified by age, the > 64 years age group had the highest adjusted percentage, rising from 37.9% in 2007–2008 to 39.23% (95% CI: 38.53%–39.94%) in 2023–2024. The 20–24 years group, which began at 17.19%, rose to 26.80% (95% CI: 25.59%–28.05%) by 2023–2024 (Figure S4B). For the least deprived group, the percentage with an HbA1c ≤ 58 mmol/mol increased from 31.2% in 2007–2008 to 41.7% (95% CI: 41.0%–42.5%) in 2023–2024. The most deprived group began at 25.1% in 2007–2008 and reached 28.9% (95% CI: 28.20%–29.52%) by 2023–2024 (Figure S4C). During the study period (2007–2008 to 2023–2024), the percentage of females with an HbA1c ≤ 58 mmol/mol ranged from 27.5% to 31.4%, while percentages for males ranged from 27.2% to 34.1%. Both sexes exhibited a general upward trend over the period (Figure S4D).

## 4 Discussion

This nationwide sequential cohort study assessed trends in glycaemic control in adults with Type 1 diabetes in England from 2007–2008 to 2023–2024. Mean HbA1c, adjusted to reflect the changing characteristics of those with Type 1 diabetes, decreased from 68.8 mmol/mol in 2007–2008 to 66.2 mmol/mol in 2023–2024. The proportion of adults with an HbA1c ≤ 58 mmol/mol increased from 32.6% to 39.1% over the same time period. However, this change was not linear; a marked improvement in glycaemic control was observed from 2020 to 2021 onwards, coinciding with the national rollout of flash glucose monitoring by NHS England. While this temporal association is notable, these data are observational and therefore cannot establish causality. Other factors, such as wider use of insulin pumps and hybrid closed‐loop systems, may also have contributed. Importantly, these gains were not evenly distributed by age, sex, social deprivation and ethnic groups, highlighting widening inequalities that warrant focused, equity‐driven public health interventions as well as technology enablement and education, perhaps particularly in Black and Mixed ethnicity populations, as well as those living in deprived areas. Recognition of these inequalities within the clinical community is essential, alongside ensuring that clinical teams have access to relevant data and are supported to develop action plans to address them.

The overall decline of 2.6 mmol/mol (68.8 to 66.2 mmol/mol) over 15 years may appear modest at the individual level, but at a population level, it is likely to be clinically meaningful, particularly given the large number of adults with Type 1 diabetes included in this study. Even small reductions in HbA1c have been associated with a lower risk of microvascular complications and may translate into important health gains when applied across an entire population. The magnitude of improvement observed in our study is broadly consistent with international evidence. A Scottish national study of 30 717 people with Type 1 diabetes reported a 4 mmol/mol reduction in median HbA1c between 2004 and 2016 (72 to 68 mmol/mol), with the improvement occurring in the latter 4 years of that study period, coinciding with national pump therapy expansion [17].

Prigge et al. observed that while England had seen improvements, the scale of reduction in median HbA1c had been less pronounced than in Scandinavian and German registries [18]. In those regions, earlier and more widespread adoption of diabetes technologies appeared to drive more substantial longitudinal gains in achieving the < 58 mmol/mol threshold compared to the trends observed in the United Kingdom.

The US T1D Exchange similarly documented that between 2016 and 2022, increased uptake of CGM and automated insulin delivery systems was associated with a significant 0.3% reduction in mean HbA1c (from 8.7% to 8.4%; *p* < 0.01). This follows a prior period (2010–2018) in which, despite increased technology use, the overall population's HbA1c rose; however, device users consistently maintained a median HbA1c approximately 0.7% lower (7.7% vs. 8.4%) than non‐users, highlighting that the scale of improvement is heavily mediated by consistent technology access [19]. The acceleration in improved glycaemic control observed in our data post‐2020–2021 coincided with NHS England's roll‐out of flash glucose monitoring and mirrors these international findings linking technology access to reductions in HbA1c.

This study showed a 13.7% relative reduction in mean HbA1c among individuals aged 20–24 years. In contrast, older adults (> 64 years) experienced minimal improvement, with only a 5% increase in the proportion achieving HbA1c ≤ 58 mmol/mol and a 2.0 mmol/mol increase in adjusted mean HbA1c. Older people generally had lower HbA1c at baseline, so there was greater scope for improvement in younger populations. These results highlight the 20–24‐year group as a major contributor to overall gains in glycaemic control, potentially reflecting earlier uptake of intensive therapies (e.g., automated insulin delivery) and greater engagement with technology‐enabled care [20, 21]. In contrast, older adults frequently encounter therapeutic inertia driven by hypoglycaemia concerns, polypharmacy and comorbidities, leading to conservative treatment intensification [22]. Survivorship bias likely contributed to overrepresentation of healthier older individuals, masking the challenges faced by frailer subgroups with more labile glycaemia [23]. A multicentre cross‐sectional analysis reported that younger adults (including those < 30 years) with Type 1 diabetes had higher median HbA1c and greater glycaemic variability than people from older age groups, highlighting systemic challenges in balancing targets with lifestyle demands [18]. Data from the Type 1 Diabetes Exchange Clinic Registry (2016–2018; *n* = 21 253) showed marked age‐related differences in glycaemic control, with mean HbA1c peaking in adolescents and young adults at approximately 9.0% (75 mmol/mol), before declining to around 7.5% (58 mmol/mol) among those aged ≥ 60 years. These findings highlight the substantially higher glycaemic burden in the younger age group [19]. A study from Northwest Iran reported better glycaemic control in older adults (≥ 60 years) with Type 1 diabetes despite higher complication rates, attributing this paradox to age‐related differences in disease acceptance, health literacy and adherence to insulin regimens [24]. This aligns with lower HbA1c in older adults found in this analysis [25]. Future research should explore whether differential adherence to therapies, socioeconomic factors, or biomarker‐guided treatment personalisation contribute to these inequalities. Policy makers must prioritise age‐tailored interventions, balancing intensive glycaemic control in younger populations.

This analysis revealed a pronounced and widening socioeconomic gradient in glycaemic control: between 2007–2008 and 2023–2024, adults in the least deprived quintile experienced a 31% increase in the likelihood of having an HbA1c ≤ 58 mmol/mol (≤ 7.5%) and an absolute reduction in mean HbA1c of 5%, whereas those in the most deprived quintile saw only a 5% rise in threshold attainment and a 1% reduction in mean HbA1c. This observation aligns with international and UK‐based research: one multicentre study reported that adults with Type 1 diabetes living in the least deprived areas had a mean HbA1c value 5.9 mmol/mol (0.5%) lower than those in the most deprived areas [26]. In addition, a decade‐long analysis of registry data from the United States and Germany demonstrated that lower socio‐economic status was consistently associated with higher HbA1c, and that widening disparities in diabetes technology use were associated with increasing HbA1c inequalities over time, particularly in the US cohort [27]. Crucially, most clinical trials of novel insulin delivery systems and adjunctive agents under‐represented deprived groups, perpetuating evidence gaps for optimal management in the populations who need it most [28]. From 2021–2022, the NDA included data on the use of wearable glucose monitoring. It showed that by March 2022, 50.4% of adults with Type 1 diabetes in England were using such devices, which were associated with lower HbA1c [28]. In 2021–2022, pump and CGM use among people living in the most deprived quintile was 44.7% and 8.5%, respectively, versus 55.8% and 13.9% in the least deprived neighbourhoods [28]. Broader social determinants, such as food insecurity, housing instability and financial stress, created additional challenges for glycaemic management [29]. A systematic review found that unemployment and low education predicted a 0.5% (5.5 mmol/mol) higher HbA1c [30].

Persistent ethnic inequalities in HbA1c were observed, with White adults showing the greatest changes, Asian adults' modest reductions and Black adults consistently having higher HbA1c, widening the gap over time. These patterns likely reflect a combination of varying access to technology and differential interactions with health services across ethnic groups, although the unequal population sizes and smaller numbers within some ethnic groups limit the ability to draw definitive conclusions. Future work should incorporate qualitative inquiry into cultural and system‐level barriers and ensure equitable inclusion in technology trials to guide targeted interventions.

Over the study period, females consistently had higher mean HbA1c and were less likely to have an HbA1c ≤ 58 mmol/mol. While the absolute differences were small, their consistency over time and large population size suggest clinical relevance and underscore the importance of considering sex‐specific factors in strategies to improve glycaemic control in adults with Type 1 diabetes [31, 32].

The fall in GP participation in the NDA around 2012–2014 was due to a period of structural reorganisation in the NHS. The dissolution of primary care trusts and other associated NHS bodies meant that local processes that had been in place to monitor and encourage participation in the NDA were lost and not immediately replaced by the new health organisations. This was identified as an issue, and a programme of engagement was put in place, which resulted in increasing participation that continued for the remainder of the period of this analysis.

This study's key strength is its large cohort drawn from the National Diabetes Audit, capturing over 130 000 adults in 2007–2008 and more than 225 000 in 2023–2024 and the use of mixed‐effects models to account for individual‐level variability in repeated HbA1c measurements while adjusting for age, sex, ethnicity and deprivation. Reporting adjusted percentage change in threshold attainment alongside relative changes in mean HbA1c enhances clinical relevance and comparability across sociodemographic groups. Nonetheless, several limitations warrant consideration. We lacked information on the co‐morbidity burden, which introduces a potential residual confounding. Excluding individuals without valid HbA1c records may have introduced selection bias if those missing data had different HbA1c measurements than those with complete data. We were not able to include data on technology use in this analysis, but further work is planned to explore this area. IMD is an area‐level measure of deprivation and may not fully capture individual‐level socioeconomic position (e.g., education, income, or occupation). Older participants may represent healthier survivors, underestimating the challenges faced as people with Type 1 diabetes age. Residual confounding by clinical complexity is likely, particularly in older adults who have greater co‐morbidity, hypoglycaemia unawareness and often higher individualised glycaemic targets. These factors are not captured in the NDA and could not be adjusted for. Relatively small sample sizes for the Black and Mixed ethnicity groups (8280 and 2760 individuals, respectively, in 2023–2024) resulted in wide CIs and so ethnic differences should therefore be interpreted cautiously, emphasising overall trends rather than year‐specific contrasts. Finally, reliance on the audit data available means we could not assess individual behavioural factors, such as adherence to treatment or psychosocial support, that critically influence long‐term glycaemic outcomes.

Despite the constraints noted above, this analysis shows that population‐level reductions in HbA1c in adults with Type 1 diabetes in England occurred between 2007–2008 and 2023–2024, but inequalities based on age, ethnicity and social deprivation have widened, highlighting the need for more granular clinical and behavioural data in future studies. The findings underscore the need for targeted, equity‐focused interventions, extending programmes such as Core20PLUS5 to adults and prioritising culturally responsive, age‐appropriate and socioeconomically inclusive care models to reduce gaps in glycaemic outcomes [33].

## Author Contributions

N.H. conceived and designed the study. M.S.K. performed the statistical analysis and drafted the original manuscript. All authors contributed substantially to the study, including interpretation of the results, drafting and critical revision of the manuscript for intellectual content and approval of the final version for publication. M.S.K. is the guarantor of this work.

## Funding

This work was funded by NHS England, Northwest London National Institute for Health and Care Research (NIHR) Applied Research Collaboration (ARC), and CW+.

## Conflicts of Interest

N.H. has received payments from AstraZeneca as a patient participating in a clinical trial. G.D.T. is supported by the National Institute for Health Research (NIHR) Oxford Biomedical Research Centre (BRC). The views expressed are those of the authors and not necessarily those of the NHS, the NIHR or the Department of Health. H.M. received speaker fees from Abbott Diabetes Care, Dexcom, Eli Lilly, Medtronic, Novo Nordisk, Sanofi and Ypsomed and support for attending meetings/travel from Ypsomed Australia and Dexcom and has received research equipment (CGM devices) from Abbott Diabetes Care, Dexcom and Medtronic. N.S. has consulted for and/or received speaker honoraria from Abbott Laboratories, AbbVie, Amgen, AstraZeneca, Boehringer Ingelheim, Carmot Therapeutics, Eli Lilly, GlaxoSmithKline, Hanmi Pharmaceuticals, Menarini‐Ricerche, Metsera, Novartis, Novo Nordisk, Pfizer and Roche; and received grant support paid to his University from AstraZeneca, Boehringer Ingelheim, Novartis and Roche outside the submitted work. J.V. was the National Clinical Director for NHS England from April 2013 to September 2023. K.K. received grants from AstraZeneca, Boehringer Ingelheim, Lilly, MSD, Novo Nordisk, Sanofi, Servier, Oramed Pharmaceuticals, Roche, Daiichi‐Sankyo and Applied Therapeutics. He has consulted for and/or received speaker honoraria from Amgen, AstraZeneca, Bristol Myers Squibb, Boehringer Ingelheim, Lilly, Novo Nordisk, Sanofi, Servier, Pfizer, Roche, Daiichi‐Sankyo, Embecta and Nestle Health Science. The other authors declare no conflicts of interest.

## Supporting information


**Figure S1:** Trends in GP Practice Participation (%) in the National Diabetes Audit, 2007–2008 to 2023–2024 in England and Wales.
**Figure S3:** (D) Trends in the adjusted percentage of adults with Type 1 diabetes in England achieving HbA1c ≤ 58 mmol/mol, stratified by sex from 2007–2008 to 2023–2024.
**Figure S4:** Trends in the adjusted percentage of adults with Type 1 diabetes in England achieving HbA1c ≤ 58 mmol/mol, among all adults with Type 1 diabetes in NDA from 2007–2008 to 2023–2024. (A) Trends in the adjusted percentage of adults with Type 1 diabetes in England achieving HbA1c ≤ 58 mmol/mol, among all adults with Type 1 diabetes in NDA, stratified by Age: 2007–2008 to 2023–2024. (B) Trends in the adjusted percentage of adults with Type 1 diabetes in England achieving HbA1c ≤ 58 mmol/mol, among all adults with Type 1 diabetes in NDA, Stratified by IMD: 2007–2008 to 2023–2024. (C) Trends in the adjusted percentage of adults with Type 1 diabetes in England achieving HbA1c ≤ 58 mmol/mol, among all adults with Type 1 diabetes in NDA, stratified by ethnicity: 2007–2008 to 2023–2024. (D) Trends in the adjusted percentage of adults with Type 1 diabetes in England achieving HbA1c ≤ 58 mmol/mol, among all adults with Type 1 diabetes in NDA, stratified by sex: 2007–2008 to 2023–2024.

## Data Availability

Data from the National Diabetes Audit and the National Diabetes Foot Care Audit may be obtained via the Data Request Service at NHS England (see https://digital.nhs.uk/services/data‐access‐request‐service‐dars).
