# Supplementary material for: Glycaemic Control by Sociodemographic Factors in Adults With Type 1 Diabetes in England: Trends From 2007–2008 to 2023–2024
Source: Diabetes Obes Metab. 2026 Jun 1;28(8):7257–66. doi: 10.1111/dom.70929 (PMC13341392; doi:10.1111/dom.70929)
Supplement: Supplementary file 1 — Figure S1: Trends in GP Practice Participation (%) in the National Diabetes Audit, 2007–2008 to 2023–2024 in England and Wales. Figure S3: (D) Trends in the adjusted percentage of adults with Type 1 diabetes in England achieving HbA1c ≤ 58 mmol/mol, stratified by sex from 2007–2008 to 2023–2024. Figure S4: Trends in the adjusted percentage of adults with Type 1 diabetes in England achieving HbA1c ≤ 58 mmol/mol, among all adults with Type 1 diabetes in NDA from 2007–2008 to 2023–2024. (A) Trends in the adjusted percentage of adults with Type 1 diabetes in England achieving HbA1c ≤ 58 mmol/mol, among all adults with Type 1 diabetes in NDA, stratified by Age: 2007–2008 to 2023–2024. (B) Trends in the adjusted percentage of adults with Type 1 diabetes in England achieving HbA1c ≤ 58 mmol/mol, among all adults with Type 1 diabetes in NDA, Stratified by IMD: 2007–2008 to 2023–2024. (C) Trends in the adjusted percentage of adults with Type 1 diabetes in England achieving HbA1c ≤ 58 mmol/mol, among all adults with Type 1 diabetes in NDA, stratified by ethnicity: 2007–2008 to 2023–2024. (D) Trends in the adjusted percentage of adults with Type 1 diabetes in England achieving HbA1c ≤ 58 mmol/mol, among all adults with Type 1 diabetes in NDA, stratified by sex: 2007–2008 to 2023–2024. [file DOM-28-7257-s001.docx]

**Supplementary**

***Figure S1*:** Trends in GP Practice Participation (%) in the National Diabetes Audit, 2007_8 –2023_24 in England and Wales

**
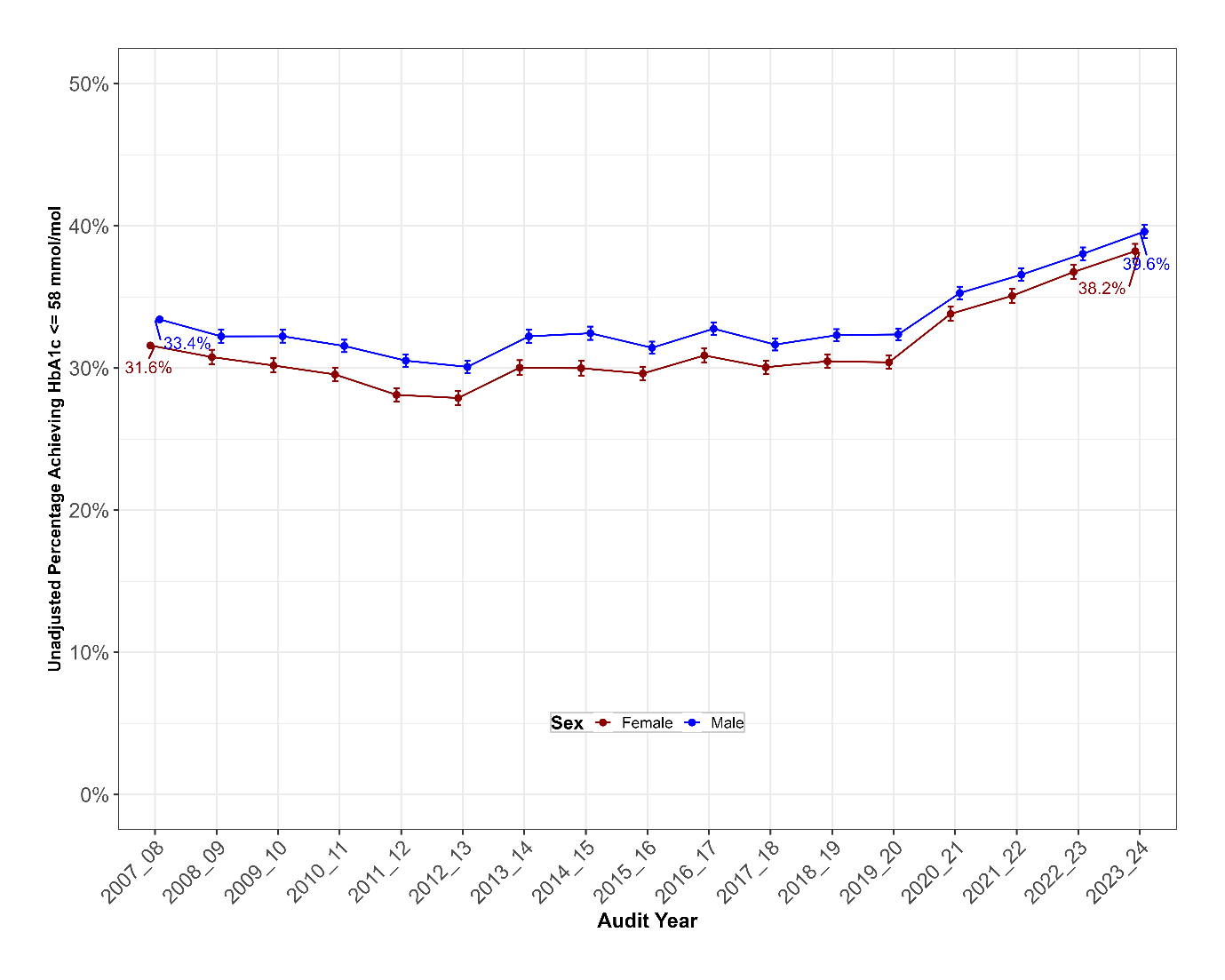
**

***Figure S3 D***: Trends in the adjusted percentage of adults with Type 1 diabetes in England achieving HbA1c ≤ 58 *mmol/mol*, stratified by sex from 2007–08 to 2023–24.

Audit period 2007–08 is the baseline reference; all estimates compare subsequent periods to baseline.

**Sensitivity analysis plots**

**
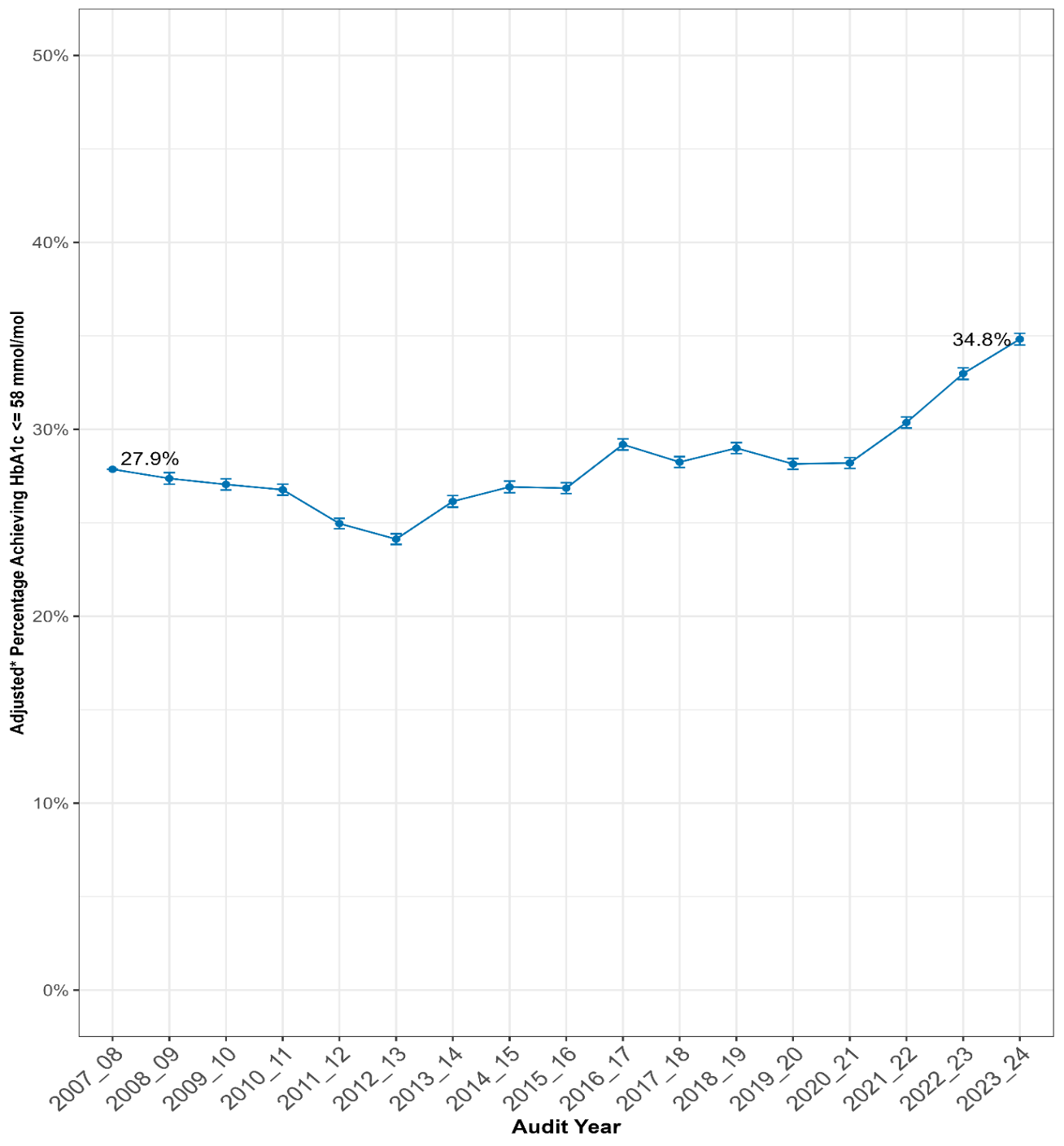
**

***Figure S4***: Trends in the adjusted percentage of adults with Type 1 diabetes in England achieving HbA1c ≤ 58 *mmol/mol*, among all adults with type 1 diabetes in NDA from 2007–08 to 2023–24

*Adjusted for Age, sex, ethnicity and deprivation

Audit period 2007–08 is the baseline reference; all estimates compare subsequent periods to baseline.

**
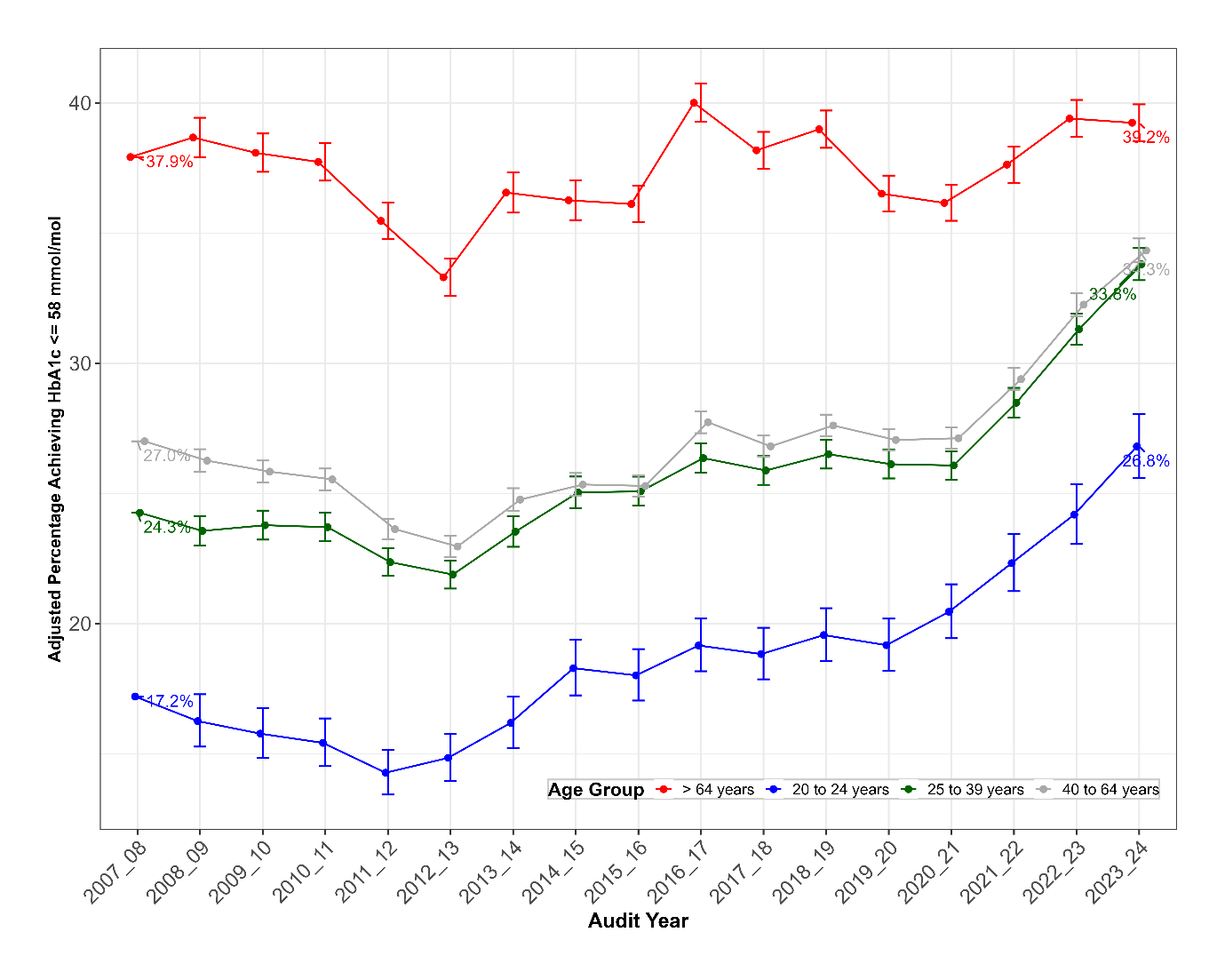
*Figure S4 A***: Trends in the adjusted percentage of adults with Type 1 diabetes in England achieving HbA1c ≤ 58 *mmol/mol*, among all adults with type 1 diabetes in NDA, Stratified by Age: 2007–08 to 2023–24

*Adjusted for sex, ethnicity and deprivation

**
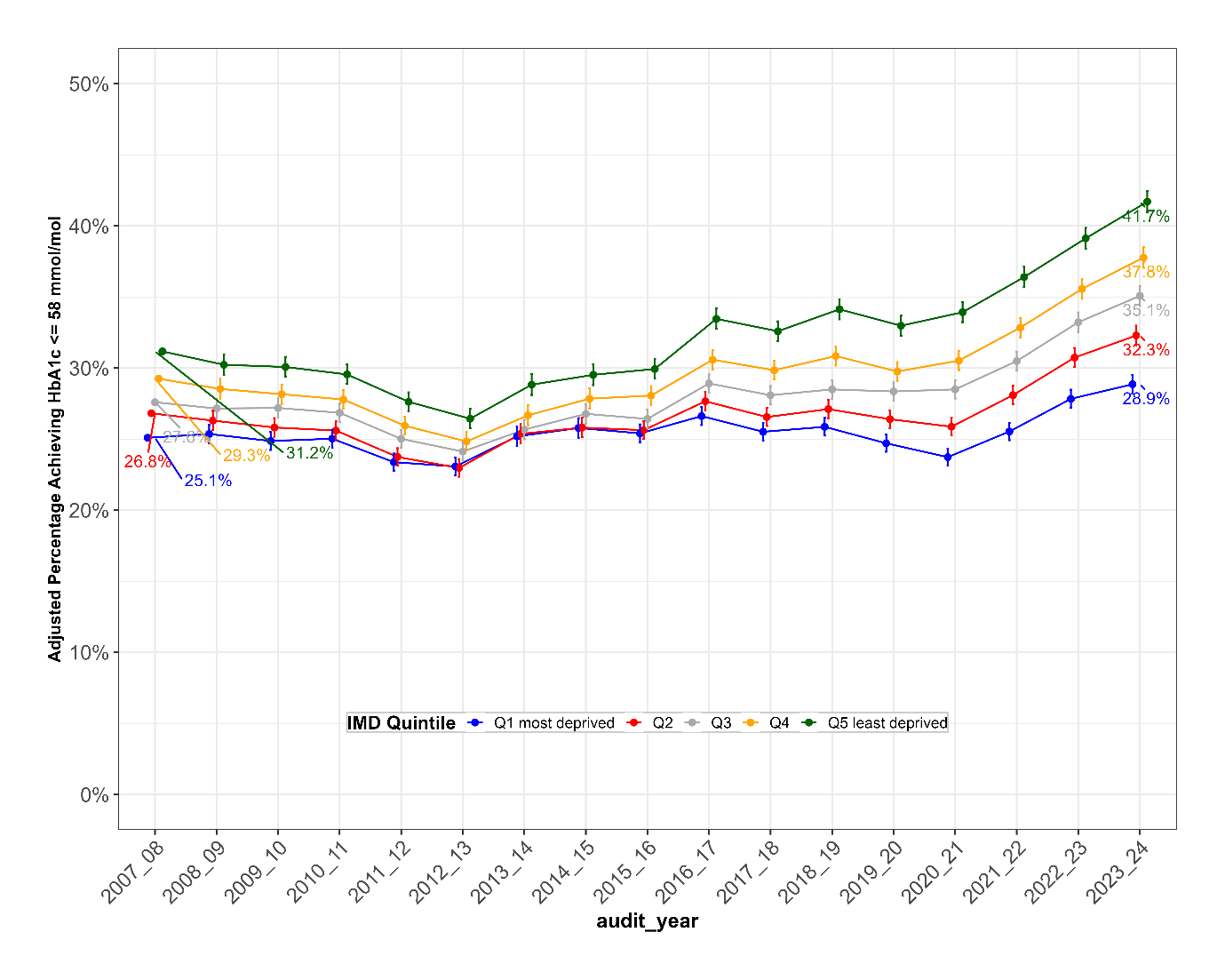
*Figure S4 B***: Trends in the adjusted percentage of adults with Type 1 diabetes in England achieving HbA1c ≤ 58 *mmol/mol*, among all adults with type 1 diabetes in NDA, Stratified by IMD: 2007–08 to 2023–24

*Adjusted for age, sex and ethnicity

**
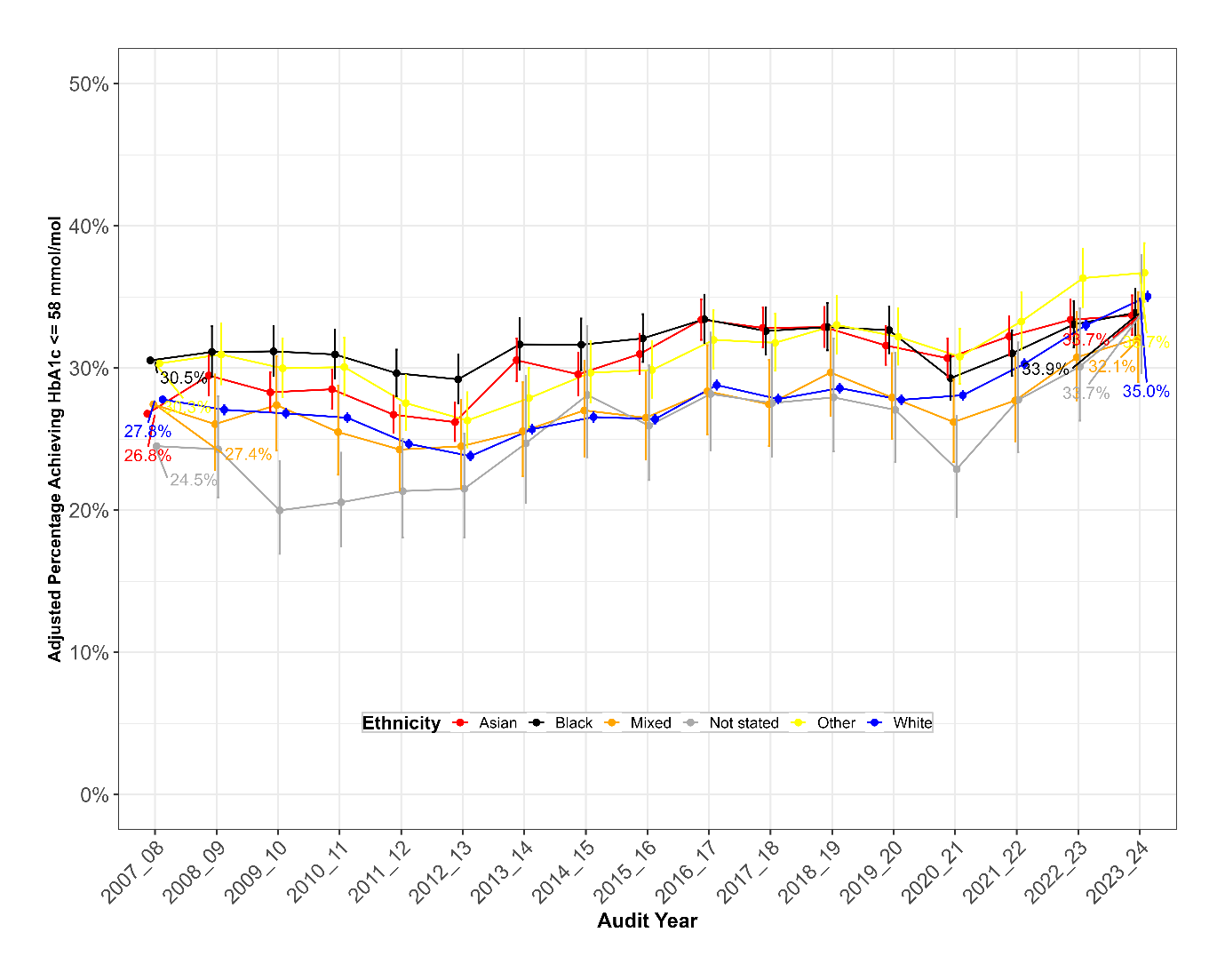
*Figure S4 C***: Trends in the adjusted percentage of adults with Type 1 diabetes in England achieving HbA1c ≤ 58 *mmol/mol*, among all adults with type 1 diabetes in NDA, stratified by ethnicity: 2007–08 to 2023–24

*Adjusted for age, sex, and deprivation

**
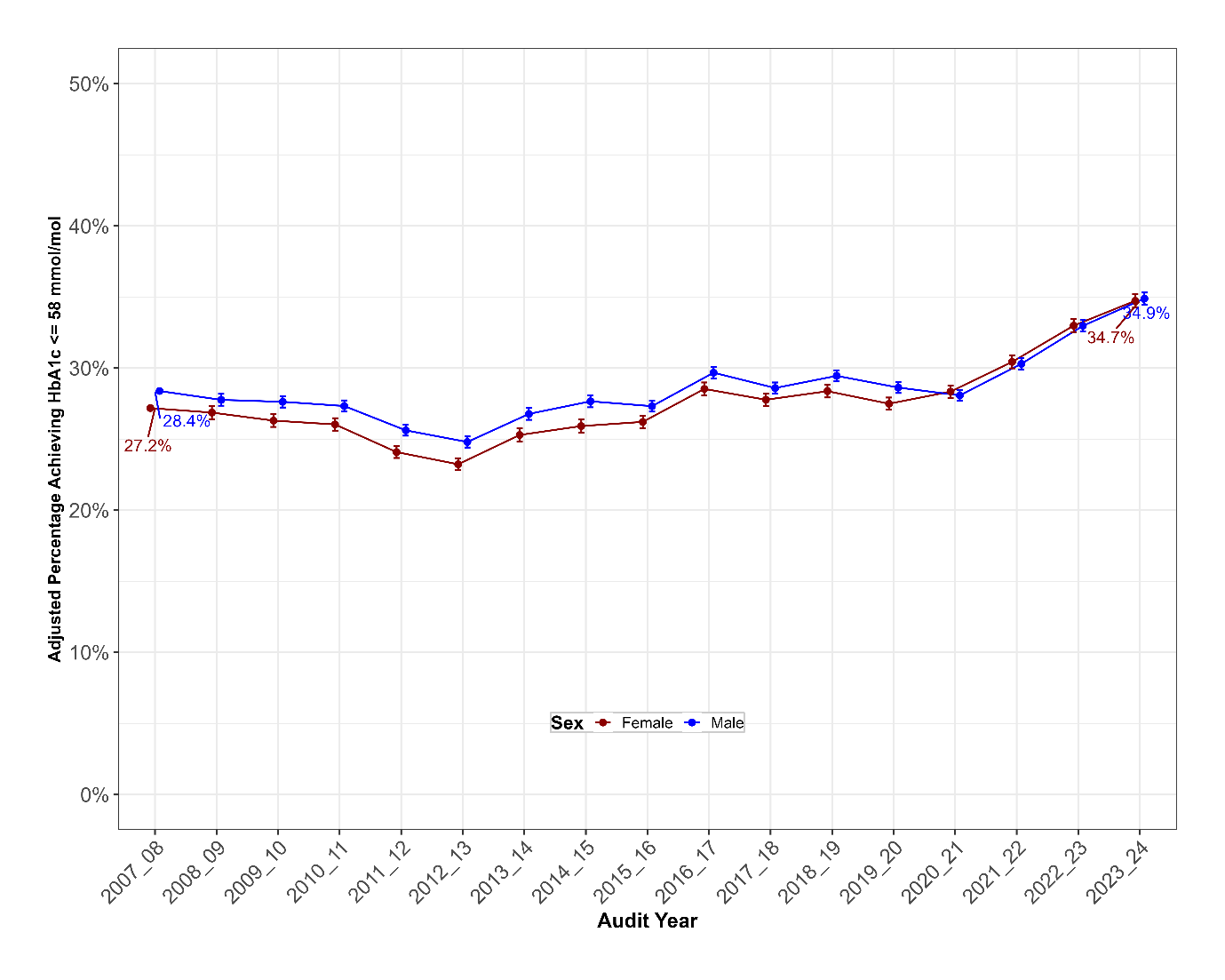
*Figure S4 D***: Trends in the adjusted percentage of adults with Type 1 diabetes in England achieving HbA1c ≤ 58 *mmol/mol*, among all adults with type 1 diabetes in NDA, stratified by sex: 2007–08 to 2023–24

*Adjusted for age, ethnicity and deprivation
